# Upper Limb Asymmetry in the Sense of Effort Is Dependent on Force Level

**DOI:** 10.3389/fpsyg.2017.00643

**Published:** 2017-04-26

**Authors:** Mark Mitchell, Bernard J. Martin, Diane E. Adamo

**Affiliations:** ^1^Rehabilitation Institute of Michigan, DetroitMI, USA; ^2^Department of Industrial and Operations Engineering, University of Michigan, Ann ArborMI, USA; ^3^Department of Health Care Sciences, Physical Therapy Program, Wayne State University, DetroitMI, USA; ^4^Institute of Gerontology, Wayne State University, DetroitMI, USA

**Keywords:** asymmetries, motor control, grasp force, feedback interactions, handedness

## Abstract

Previous studies have shown that asymmetries in upper limb sensorimotor function are dependent on the source of sensory and motor information, hand preference and differences in hand strength. Further, the utilization of sensory and motor information and the mode of control of force may differ between the right hand/left hemisphere and left hand/right hemisphere systems. To more clearly understand the unique contribution of hand strength and intrinsic differences to the control of grasp force, we investigated hand/hemisphere differences when the source of force information was encoded at two different force levels corresponding to a 20 and 70% maximum voluntary contraction or the right and left hand of each participant. Eleven, adult males who demonstrated a stronger right than left maximum grasp force were requested to match a right or left hand 20 or 70% maximal voluntary contraction reference force with the opposite hand. During the matching task, visual feedback corresponding to the production of the reference force was available and then removed when the contralateral hand performed the match. The matching relative force error was significantly different between hands for the 70% MVC reference force but not for the 20% MVC reference force. Directional asymmetries, quantified as the matching force constant error, showed right hand overshoots and left undershoots were force dependent and primarily due to greater undershoots when matching with the left hand the right hand reference force. Findings further suggest that the interaction between internal sources of information, such as efferent copy and proprioception, as well as hand strength differences appear to be hand/hemisphere system dependent. Investigations of force matching tasks under conditions whereby force level is varied and visual feedback of the reference force is available provides critical baseline information for building effective interventions for asymmetric (stroke-related, Parkinson’s Disease) and symmetric (Amyotrophic Lateral Sclerosis) upper limb recovery of neurological conditions where the various sources of sensory – motor information have been significantly altered by the disease process.

## Introduction

Asymmetries in upper limb performance are associated with ones’ hand preference and handedness is an established indicator of intrinsic cerebral asymmetry (Liu et al., 2009). Support for these differences have been evidenced at functional (Kim et al., 1993; Triggs et al., 1999) and structural (Kim et al., 1993; Amunts et al., 1996; Babiloni et al., 2003) levels and have emerged through the preferential use of information to control movement (Sainburg, 2005). Furthermore, asymmetry in position sense (Adamo and Martin, 2009; Scotland et al., 2014), movement sense (Martin and Adamo, 2011) and sense of effort (Scotland et al., 2014) and, the provision of analytical models (Adamo and Martin, 2009; Martin and Adamo, 2011) have shown that sensorimotor asymmetries reflect a difference in gain (information input-motor output relationship) relative to each hand/hemisphere system.

The gain concept provides a foundation for understanding the existence of asymmetries in upper extremity tasks as it takes into account the relationship between sensory input and motor output which is unique to each hand/hemisphere system. In a closed loop system, the sensory-motor feedback loop includes information from muscle spindles, Golgi tendon organs, and cutaneous sensory receptors, central processing components associated with corpus collosum transfer functions, motor commands and muscle force generation. Upper extremity matching tasks whereby sensory input information issued from one hand/hemisphere system is used to generate a matching response with the opposite hand have provided evidence of a gain higher for the non-dominant than dominant side in right handers (Adamo and Martin, 2009; Martin and Adamo, 2011; Adamo et al., 2012; Scotland et al., 2014).

We recently showed that sources of information (visual, efferent, or afferent) and the complex interactions between them differ for the dominant and non-dominant hand (Scotland et al., 2014). We also suggested a preferential feedforward and feedback control for the right and left hand, respectively (Srinivasan and Martin, 2010; Scotland et al., 2014). In right-handed individuals the sense of force effort asymmetry was associated with intrinsic anatomical, neurophysiological and musculoskeletal differences inherent to each hand-hemisphere system and a peripheral difference associated with muscle strength (Adamo et al., 2012). Asymmetry was expressed by right hand overshoots and left hand undershoots when the contralateral hand provided the reference force to be matched. These directional differences were larger when the dominant right hand was at least 5% stronger than the left hand and reduced when grip strength was equivalent between the hands. They tended to be reversed when the left hand was stronger than the right. These findings supported the gain concept as the asymmetry in force matching was shown to be a consequence of both a difference in cortical representations and motor components.

Furthermore, the each source of information (visual, efferent, or afferent) used to establish the reference force level and, thus its internal representation cannot be overlooked since each type of information is different and requires different perceptuo-motor transformations. Indeed, asymmetry of bilateral finger force matching is suppressed when visual feedback represents the output force of the reference dominant hand. In contrast, asymmetry persists with a visual feedback of the reference non-dominant hand (Henningsen et al., 1995). Further, Scotland et al. (2014) showed the absolute error was generally larger when visual feedback of the 20% maximum voluntary contraction (MVC) reference force was provided and more pronounced when matching the right hand reference with the left hand, suggesting that the ability of the dominant arm to use visual information to control movement (Sarlegna and Sainburg, 2009) may also subsist in force control.

Since the sense of effort appears to be asymmetric and different sources of information (visual, efferent, or afferent) used to establish the reference force play a significant role in force reproduction/control, the goal of the present study was to investigate the dependence of this asymmetry on two different reference force levels (20 and 70% of MVC) and when the right hand was stronger than the left hand. By comparing two different force levels, the intrinsic asymmetries unique to each hand-hemisphere system will be better understood since force matching does not appear to be a linear process (Jones and Hunter, 1982) and variability increases with the force level (Schmidt et al., 1979) which is more pronounced for the right dominant than left non-dominant hand (Yao et al., 2000).

Using a force matching paradigm will offer new insights into the management of individuals who suffer neurological injuries, such as unilateral stroke or Parkinson’s Diseases (Yust-Katz et al., 2008), known to result in asymmetric upper extremity changes in sensorimotor processing and performance. Further, provision of visual feedback while establishing the reference force aligns with current treatment approaches whereby visual feedback is typically provided when performing an exercise or activity to improve strength and function (Campenella et al., 2000).

From a clinical perspective, baseline and follow up measures of hand grip strength are based on ones’ maximum grip. Improvements in maximum grip strength may indicate that a strength training intervention has been effective, for example. Since, maximum grip strength is a common clinical measure we used a second force level of 70% MVC to better align our paradigm with existing clinical measures without introducing the risk of fatigue. A change in maximum grip strength, from a higher to lower value is indicative of the progressive decline in muscle strength that differs as a function of age and/or the onset of musculoskeletal and neurological impairments (Beck et al., 1999; Visser et al., 2003). The rate of change in the decline of hand grip strength is different for the right and left hand (Chatterjee and Chowdhuri, 1991), will be more evident at higher than lower force levels, and differs for men and women (Vianna et al., 2007). Given the existence of inherent hand – hemisphere differences in the control of grasp force and grip strength, it is reasonable to justify the use a contralateral force matching paradigm to investigate asymmetric upper extremity changes in sensorimotor processing and performance.

To further build on existing data with the intention of translating our research into clinical applications, the following hypotheses were tested: (a) the magnitude of force matching error will be greater for the 70 than 20% MVC reference force level and, (b) based on our previous results (Scotland et al., 2014) indicating a role of proprioceptive information in left hand force perception we may assume that interactions between efferent and afferent information should be stronger for the left than right hand system and for higher than lower force exertions. To test these hypotheses, matching a 20 and 70% (MVC) grasp force with the contralateral hand was investigated in a group of strongly right-handed males with a stronger right than left hand to avoid issues associated with handedness heterogeneity (Hoom et al., 2012) and interaction effects associated with diverse hand strength differences (Adamo et al., 2012).

## Materials and Methods

### Participants

Eleven right-handed males (mean age ± SD: 24.9 ± 4.9 years) with a handedness laterality index of 1.0, as assessed by the modified Edinburgh Handedness Inventory (Williams, 1986) participated in the experiment. All participants demonstrated a stronger right than left grip strength and were free from any upper limb neurological or musculoskeletal conditions that might compromise task performance. Exclusion criteria included a long-standing history of highly skilled motor activity such as dancing or playing a musical instrument. This study was carried out in accordance with the recommendations of Wayne State University Human Investigation Committee with written informed consent from all subjects. All subjects gave written informed consent in accordance with the Declaration of Helsinki. The protocol was approved by the Wayne State University Human Investigation.

### Experimental Procedures

Prior to the experiment, MVC for each hand was measured using a Jaymar^TM^ dynamometer in a standard posture with the elbow flexed at 90° and the wrist in slight extension. The handle of the dynamometer adjusted to the anthropometry of each individuals’ hand. Participants were instructed to increase force gradually over 2 s, then sustain their maximum exertion for an additional 2 s before releasing their grasp on the device. The average of two trials determined grip strength. To avoid fatigue, MVC exertions were alternated between hands and a 1-min rest break separated subsequent exertions. A 5 min rest period followed the MVC measurements. This measure was repeated at the end of the experiment to ensure fatigue did not interfere with performance.

### Experimental Set-up

**FIGURE 1:**
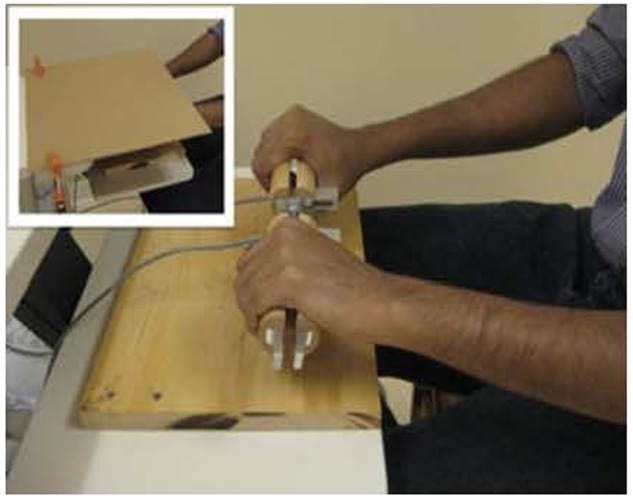
**Custom designed grasp devices composed of a split aluminum force transducer embedded with strain gauges and equipped on each side with semi-circular wooden handles (4.0 cm in radius, 13 cm in length).** The grasp devices were held horizontally by a coupling support fixed to the table. Insert shows that hands were masked during the experiment.

Participants were seated with elbows positioned at 120° flexion, wrists extended ≈30° and forearms pronated to grasp the instrumented devices placed symmetrically in front of them at midline (see **Figure 1**). A visual display placed 58.4 cm away from the participant’s eyes provided the visual feedback corresponding to right and left hand 20 and 70% reference hand forces. The force scale was calibrated to the respective 100% MVC for each hand and a horizontal cursor indicated the 20 and 70% MVC_REF_ on the visual display.

Prior to testing, each participant was provided with a brief training session that included 2–3 trials per hand to ensure task requirements were clearly understood and to demonstrate an ability to gauge the amount of force needed to reach the displayed reference force level. Participants were instructed to grasp the device and gradually increase the amount of force exerted on the handle until they reached the target/reference force level. Once the target force level was reached, it was required to maintain a steady grasp by keeping the reference force between two horizontal cursors that represented ± 5% of the force level. It was important participants learned how to establish the reference force and to remember how much force they exerted to reach the target since visual feedback was not provided when they performed the match. Test trials were administered after the training session.

Participants completed a Contralateral Matching task. For this task, the 20 and 70% MVC reference force (20, 70% MVC_REF_) exerted by one hand (right or left) with visual feedback was matched with the opposite hand without visual feedback, 2 s after the reference grasp force was released and the reference force profile returned to baseline. Reference hand (R, L) and force level (20, 70%) were counterbalanced across participants using a randomized block experimental design; however, trials pertaining to the same hand and force level combinations were performed consecutively. Trials were repeated if the participant did not achieve or exceeded the reference force by ±5%; hence, an average of 4 initial trials across all conditions for all participants were excluded and repeated. All participants completed three trials for which they matched the 20 or 70% MVC_REF_, with either the right or left hand. This resulted in a total of 12 trials for each participant. To ensure fatigue did not interfere with performance, maximum grip strength measures taken pre/post testing were performed. Participants were offered rest breaks as needed.

### Data Acquisition and Processing

The analog signals from both force transducers were digitized at 100 Hz and low pass filtered (4th order Butterworth, zero phase lag, 6 Hz cut off frequency) using customized software (LabVIEW, National Instruments). Reference and matching forces were computed by averaging the force signal over the most stable region (<5% variation over a 2 s period) of the force profile. The average % target difference between the reference and matching force ([|F_ref_ – F_match_| /F_ref_]^∗^ 100) constituted the relative error. Constant errors were normalized to % MVC and calculated by averaging the difference between the matching and reference forces. A positive value indicated an overestimation and a negative value indicated an underestimation. Force steadiness was determined by calculating the coefficient of variation (CV = [SD/mean] × 100) corresponding to the 2 s period of the most stable region of the matching force profile, also used for the measure of the relative error.

### Data Analysis

A two-way analysis of variance (ANOVA) with repeated measures was conducted to test for main and interaction effects for matching hand (R, L) and force reference level (20%, 70%) for each dependent variable: relative error (RE), constant error (CE), and force steadiness quantified as the (CV). To determine which factors influenced main and interaction effects, *post hoc* comparisons, based on Bonferroni adjustments for multiple comparisons were used. Force matching errors for RE, CE, and force steadiness were reported as the mean (M) ± standard error (SE). Paired sample *t-*test were used to determine maximum grip strength differences between hands. Grip strength measurements are reported as the mean (M) ± standard deviation (SD). Significance was set at *P* ≤ 0.05.

## Results

### Grip Strength

Right hand pre (489.4 ± 58.8 N) and post (473.6 ± 62.7 N) experiment MVCs were not significantly different [*t*(10) = 2.1; *P* = 0.06] nor were left hand pre (450.1 ± 75.5 N) and post (442.2 ± 67.7N) MVCs [*t*(10) = 0.37; *P* = 0.71]. These comparisons ensure that fatigue was not a confounder. However, the MVC was significantly greater [8.0%, *t*(10) = 2.6; *P* = 0.024] for the right (489.4 ± 58.8 N) than left (450.1 ± 75.5 N) hand, which satisfied the inclusion criteria, and remained significantly greater post-testing [*t*(10) = 2.3; *P* = 0.042].

### Relative Error

**FIGURE 2:**
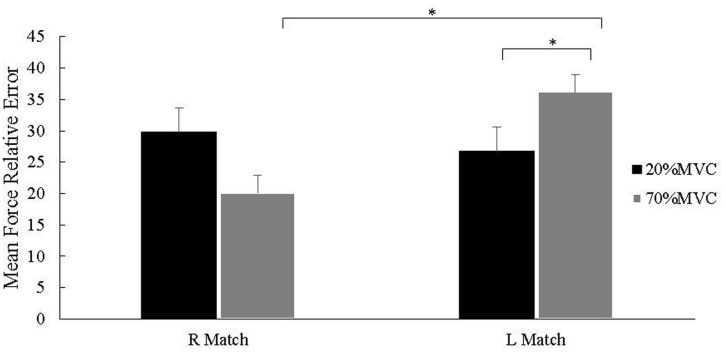
**Force mean relative error (RE) (% MVC ± SE) for right and left hand matching.** REs were not significantly different for right and left hand matching at the 20% MVC_REF_ (*P* = 0.23). At the 70% MVC_REF_ RE was smaller for right than left hand matching (^∗^*P* < 0.01). For right hand matching, RE was not significant between the 20% than the 70% MVC_REF_ condition. For left hand matching, RE was smaller for the 20% than at the 70% MVC_REF_ condition.

When the reference and corresponding matching forces were normalized to each hand % MVC, the two-way ANOVA (hand, force level) showed a significant hand × force interaction effect (*F*_(1,32)_ = 6.6, *P* = 0.015). *Post hoc* analyses indicated that the RE was not significantly different (*P* = 0.23) between right (29.8 ± 3.8%) and left hand matching (26.8 ± 2.8%) for the 20% MVC_REF_; whereas for the 70% MVC_REF_ RE was significantly smaller (*p* = 0.01) for right (20.1 ± 2.8%) than left hand matching (36.2 ± 3.0%). RE for right hand matching was not significantly different (*P* = 0.056) between the 20 and 70% MVC_REF_ (29.8 ± 3.8 and 20.1 ± 2.8%, respectively). In contrast, RE was significantly smaller (*P* = 0.004) for left hand matching at the 20% MVC_REF_ (26.8 ± 2.8%) than at the 70% MVC_REF_ (36.2 ± 3.0%). These results are illustrated in **Figure 2**.

### Constant Error (% MVC)

**FIGURE 3:**
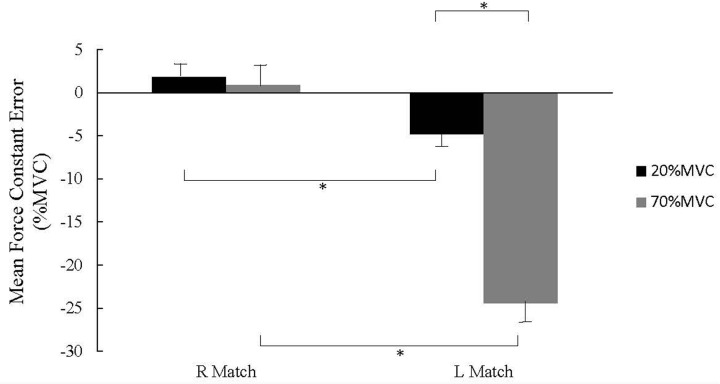
**Force mean constant error (CE) (% MVC ± SE) for right and left hand matching.** Right hand overshoots were significantly different from left hand undershoots for each reference force level (^∗^*P* < 0.01). Right hand CEs were not significantly different between 20 and 70% MVC_REF_ conditions. Left hand CEs were significantly different between the 20 and 70% MVC_REF_ conditions.

When forces were normalized to % MVC, the two-way ANOVA (hand, force level) showed a significant hand × force interaction (*F*_(1,32)_ = 47.8, *P* < 0.001). Right hand overshoot tendencies (.13 ± 2.2% MVC) and left hand undershoots (-24.4 ± 2.1% MVC) were significantly different (*P* < 0.001) at the 70% MVC_REF_. Significant differences (*P* < 0.001) between right hand overshoots (1.8 ± 0.87% MVC) and left hand undershoots (-4.8 ± 0.67% MVC) were also found at the 20% MVC_REF_, as illustrated in **Figure 3**.

For right hand matching, the 20 and 70% MVC_REF_ matching (1.8 ± 0.87% MVC and 0.13 ± 2.2% MVC, respectively) were not significantly different (*P* = 0.465). Corresponding differences at the 20 and 70% MVC_REF_ for left hand matching (-4.8 ± 0.67% MVC and -24.4 ± 2.1% MVC, respectively) were significant (*P* < 0.001), as also shown in **Figure 3**.

### Correlation

**FIGURE 4:**
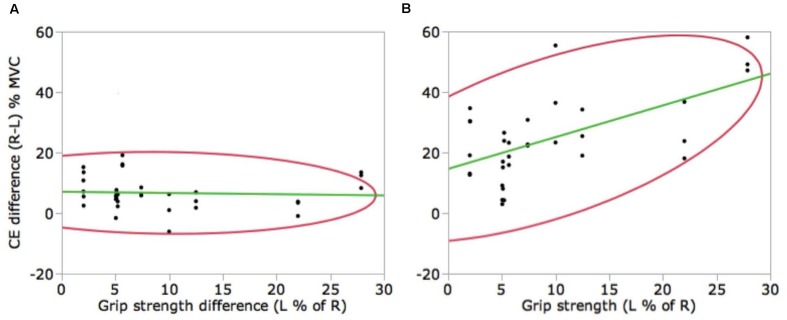
**Correlation between the difference in right and left-hand CE (in % MVC) and the difference in hand strength (in % MVC) for the 20% MVC** **(A)** and 70% MVC **(B)** reference force levels. Correlation is significant only for the 70% MVC_REF_ (*P* < 0.001).

The difference in CE between the right and left hand was correlated with the difference in grip strength only for the 70% MVC_REF_ (correlation coefficient *r* = 0.58, *P* = 0.0004), as illustrated in **Figure 4**. Taken together, this correlation and above results indicate that the greater the difference in grip strength the larger the left hand matching undershoot when the reference force is large.

### Force Steadiness

The two-way ANOVA showed a significant difference in force steadiness for matching hand (*F*_(1,32)_ = 4.5, *P* = 0.04) and reference level (*F*_(1,32)_ = 5.4, *P* = 0.026). However, interaction effects “matching hand × force level” were not significant (*P* > 0.05). Variability was significantly greater (*P* = 0.04) for right (3.0 ± 0.78) than left hand (1.1 ± 0.35) matches. Further, matching at the 20% MVC_REF_ (1.0 ± 0.50) was significantly less variable (*P* = 0.026) than matching at the 70% MVC_REF_ (3.1 ± 0.70).

## Discussion

The results showed that differences in RE between matching hands were dependent on the reference force level as they were significant only for the 70% MVC_REF_. Significant differences in CE between hands were found for both 20 and 70% MVC_REF_ forces.

### Asymmetry Factors

In right-handed individuals, hand strength differences, laterality (a measure of hand preference), intrinsic hemisphere-hand differences and the availability of visual feedback may account for matching error asymmetry. When comparing the present and previous results concerning strongly right handed males and females (Adamo et al., 2012) the following similarities are noted: (1) although grip strength was about 140N greater for each hand, the range between 5 and 28% and the average 14.5% difference in grip strength between hands were similar; (2) the laterality indices of 1.00 vs. 0.81 are high; and (3) the 20% MVC_REF_ was tested in both studies. Of interest in the present study, was to determine whether this asymmetry was modified when the force to be matched was substantially larger and tested in a group of males only. Indeed, it was found that a higher reference force of 70% MVC compared to 20% MVC differentially influenced the matching response.

### Hand Strength Differences

Although directional differences in constant error were similar to previous findings for the 20% MVC reference force (Adamo et al., 2012), the magnitude of differences found at the 70% MVC primarily resulted from the large undershoot with left hand matching. In the present case, this difference increased from 1.6% at the 20% MVC_REF_ to 5.7% at the 70% MVC_REF_, which corresponds to a 3.5 fold increase. In other words, the higher the reference force the greater the relative proportion of its maximal strength the weaker hand must exert to match the reference established by the stronger hand. When the reference force is low, the influence of the small between-hands relative difference is integrated with the visual information (force output). However, the larger between-hands relative difference associated with a large reference force may exacerbate the incongruence between the visual force level and the internal force representation. Indeed, we previously argued that the association between the force exerted and its internal representation is better when the effort information is based on visual information representing the exertion outcome than an internal representation based on the association with the efference copy or efference copy + proprioceptive feedback, depending on which matching hand is considered (Scotland et al., 2014). Thus, discrepancies in the elaboration of internal representation (association between force exerted and perception of effort) are a likely component of incongruence evoked above. Hence, this incongruence induces a reemergence of the influence of hand strength difference at the high level of reference force. Support for this phenomenon is suggested by the significant increase in CE difference with hand strength difference (**Figure 3**). The exacerbation of the asymmetry in matching is primarily due to left hand undershooting (**Figure 3**) and greater strength differences between hands, with the left substantially weaker than the right.

Several factors contribute to this large asymmetry. First, force matching does not appear to be a linear process as also suggested by earlier results from Jones and Hunter (1982). Second, force variability is known to increase with the force level (Schmidt et al., 1979) which is more pronounced for the right dominant hand, as variability is larger with MU synchronization (Yao et al., 2000) and synchronization increases with force level (Schmied and Descarreaux, 2010). Third, the higher gain for the left than right hand-hemisphere system (Martin and Adamo, 2011; Adamo et al., 2012) also predicts an increase in asymmetry with force level since a larger input to the left hand system will result in a larger undershoot due to the multiplying effect of the gain. In other words, for a high-level right hand reference force, the perception of effort equality (between the right reference and left matching hand) will occur for a proportionally lower left hand matching force when compared to a low-level right hand reference force.

Such asymmetry is coherent with similar findings reported previously (Adamo et al., 2012) when visual feedback of the reference force was not available. However, in the visual condition here, the asymmetry is more pronounced, particularly for the 70% MVC_REF_. This, primarily, results from a large undershoot for the left hand match which appears to be enhanced by greater grip strength differences between the hands when the reference force is high. In addition, the right and left-hand difference in CE was significantly correlated with right and left-hand difference in hand strength for the 70% MVC reference force level.

### Clinical Implications

Studies that investigate outcome measures using healthy cohorts are often applied to clinical populations in an effort to improve functional performance and quality of life. In the present study, we used males who were not only strongly right handed but also showed a stronger right than left hand to build on our existing work investigating asymmetries in upper extremity motor performance and its specificity according to gender. It was noted that % MVC force level resulted in increasing the magnitude of the directional differences in force matching. This was reported to result from hand/hemisphere differences in central processing (internal effort representation) at the cortical level and differences in right and left hand grip strength.

In a clinical setting the contribution of each hand to the performance of bimanual tasks is rarely observed yet, rather, focuses on the impaired/weaker side. Findings, here, suggest that both hands would benefit from participating in training programs since it is clear that right and left hand differences in force matching performance exists in healthy populations which implies that these inherent differences (strength, force control, and perception) may serve as a foundation for adapting injury recovery rehabilitation. A contralateral force matching paradigm requires individuals to use force information from one hand/hemisphere system to reproduce a match with the opposite hand, thus offers additional insight into the sense of effort for each hand system, force memory and inter-hemispheric transfer of information. In addition, the required force output for a task is not typically calibrated to ones’ maximum force generating capacity, which brings into question the benefit of treatment interventions. Varying the muscular effort demand to perform a task should be relative to an individuals’ right and left maximum hand grip strength to optimize recovery. Furthermore, using visual feedback to enhance learning associated with force generation tasks may contribute differentially to right and left hand improvements in processing force related information.

Lastly, the force-matching paradigm presented here translates easily to clinical settings. Such tasks offer a higher level of precision to monitoring strength changes in each hand, between hands and their associated central processing components, which is critical to monitor during the progression and/or recovery of an injury or disease. Functional benefits are expected to be driven by activation of plasticity of the circuitry/pathways and cortical areas (Hallett, 2001) involved in required activities, as well as at the level of the muscles implicated. The aim of a rehabilitation intervention would be to restore an expected natural asymmetry and not an equivalence between the two limbs, especially when one is used to retrain the other.

## Conclusion

The interaction between sources of information in the representation of force (efferent copy and proprioceptive) and hand strength differences appear to be hand/hemisphere system dependent. This underlines the differential and/or conflicting use of information used by each system to build the internal representation of the force and to execute the control of force. Exacerbation of asymmetry in sense of effort at the high reference force confirms the system gain difference hypothesis and has significant implications for management of clinical populations.

## Author Contributions

It is acknowledged that all authors contributed to the following criteria: substantial contributions to the conception or design of the work (DA, BM); and/or the acquisition (MM), analysis (DA, MM, BM), and data interpretation for the work (DA, BM). Participant recruitment, participant scheduling and all IRB requirements to obtain informed consent (MM). Drafting the work or revising it critically for important intellectual content (DA, BM). Final approval of the version to be submitted for consideration of publication (DA, MM, BM). Agreement to be accountable for all aspects of the work in ensuring that questions related to the accuracy or integrity of any part of the work are appropriately investigated and resolved.

## Conflict of Interest Statement

The authors declare that the research was conducted in the absence of any commercial or financial relationships that could be construed as a potential conflict of interest.
